# Prevention is the key

**DOI:** 10.1093/ehjcvp/pvae090

**Published:** 2025-01-11

**Authors:** Stefan Agewall

**Affiliations:** Editor-in-Chief, Institute of Clinical Sciences, Karolinska Institute of Danderyd, Stockholm, Sweden

**Figure 1:**
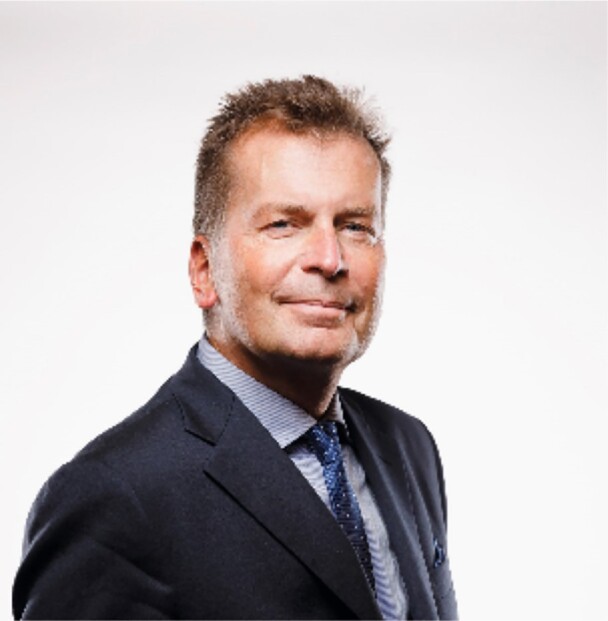


Should patients with acute coronary syndrome (ACS) and a transient episode of new-onset atrial fibrillation (AF) be given anticoagulant treatment?^1^^,^^2^ AF is one of the most prevalent complications observed in patients with ACS.^3–5^ For this last issue of the year, Dr Sorrentino and co-workers from Italy have examined the overall risk of long-term adverse events of a transient episode of new-onset AF in patients with ACS. The authors used 7 observational studies, comprising 151 735 patients; 6597 experienced transient new-onset AF, which was associated with an increased risk of ischaemic stroke, recurrent AF, or all-cause mortality. The authors concluded that occurrence of transient new-onset AF is associated with an elevated long-term risk of stroke, recurrent AF, and all-cause mortality in patients with ACS. The paper is followed by an editorial by Dr Lip and co-workers, who concluded that future prospective studies should also include long-term monitoring to understand how AF relates to outcomes, and whether it is AF burden or AF density being the more important consideration. The clinical validation of wearable devices has the potential to see them being integrated into monitoring pathways.

Cancer-associated thrombosis (CAT) is a primary cardiovascular (CV) complication encountered during cancer treatment, and venous thromboembolism (VTE) could be the most prevalent CAT. The current guidelines in the field of oncology, including those laid by the National Comprehensive Cancer Network, American Society of Clinical Oncology, and International Initiative on Thrombosis and Cancer, recommend providing anticoagulation therapy for a minimum of 6 months for cancer-associated VTE.^6^^,^^7^ Incidence of arterial thromboembolism (ATE) among ambulatory cancer patients varies by primary tumour site. In a paper from Canada, Dr Xu and co-workers conducted a systematic analysis, and included randomized trials comparing prophylactic anticoagulation to no anticoagulation among ambulatory cancer patients who initiated tumour-directed systemic therapy. The authors included 10 randomized controlled trials with 9875 patients with follow-up ranging from 3.3 to 68 (median 6.6) months. While prophylactic anticoagulation did not reduce ATE risks overall, it conferred a protective effect among pancreatic cancer patients, without a detectable increase in major bleeding. The authors concluded that prophylactic anticoagulation did not reduce ATE risks among ambulatory cancer patients overall. However, we observed lower incidence of ATE among pancreatic cancer patients randomized to receive anticoagulation.

All patients with an ACS should be treated with lipid-lowering therapy. Preferably with a high dose but we know that some patients are given lower doses of statins due to fear for side effects. In these cases, it is possible to prescribe a less aggressive statin treatment in combination with another drug,^8^^,^^9^ such as bempedoic acid^10^ and ezetimibe.^11^ Other lipid-lowering drugs are subtilisin–kexin type 9 inhibitors (PCSK9),^12^ fibrates,^13^ CSL112 [apolipoprotein A–I (human)],^14^ and cholesterol ester transfer protein inhibition.^15^ Dr Hong and colleagues from Korea investigated whether the beneficial effects of combination lipid-lowering therapy extend to patients treated with atorvastatin, not rosuvastatin, in daily clinical practice. The authors concluded that, in clinical practice, a combined lipid-lowering approach utilizing ezetimibe and moderate-intensity atorvastatin was correlated with favourable clinical outcomes, drug compliance, and a reduced incidence of new-onset diabetes requiring medications in patients treated with drug-eluting stents implantation.

Hypertension, dyslipidaemia, and diabetes are the most prevalent risk factors leading to CV morbidity and mortality.^16^ Dr Böhm and co-workers from Germany aimed to assess whether a single pill concept (SPC) is superior to a multi-pill concept (MPC) in reducing CV events, all-cause death, and costs in CV patients. Anonymized medical claims data covering 2012–18, including patients with hypertension, dyslipidaemia, and CV diseases who started a drug therapy either as SPC or identical MPC, were analysed after 1:1 propensity score matching. Hospitalizations with predefined CV events, all-cause mortality, and costs were studied in 25 311 patients with SPC and 25 311 patients with MPC using incidence rate ratios and non-parametric tests for continuous variables. The study group reported that SPC was associated with lower incidence rates of CV events, time to CV events, and all-cause death, and is superior regarding pharmacoeconomic parameters and should therefore become standard of care to improve outcomes and reduce healthcare costs. The study is followed by an editorial by Dr Penson and Panach.

Guidelines recommend initiation of dual combination antihypertensive therapy, preferably single-pill combination (SPC), in most patients with hypertension.^17^ Monte Carlo simulation was applied to 1.1 million patients qualifying for dual combination therapy from a previously conducted retrospective analysis of clinical practice, hospital statistics, and national statistics in the UK. Dr Coca *et al*. provide 10-year Kaplan–Meier event rates for the primary endpoint representing a composite of non-fatal myocardial infarction, non-fatal stroke (ischaemic or haemorrhagic), non-fatal heart failure hospitalization, or CV death. The authors claim this study represents the first to investigate guidelines-based treatment in hypertensive patients and demonstrates the opportunity for considerable risk reduction by ensuring recommended dual therapy in clinical practice, particularly in the form of SPC with high persistence, relative to no treatment or monotherapy.

The recent Randomized Evaluation of Decreased Usage of Beta-Blockers after Acute Myocardial Infarction (REDUCE-AMI) trial^18^ showed that long-term use of β-blockers in patients with acute myocardial infarction (AMI) and preserved left ventricular ejection fraction (≥50%) did neither reduce the risk of subsequent CV endpoints including death compared with no β-blocker use nor increase hospitalizations for adverse events.^19^ Other similar studies are ongoing^20^ and registry studies have shown that discontinuation of β-blockers 1 year or later after a myocardial infarction without heart failure was not associated with increased serious adverse events.^21^ Observational data on the possible influence of β-blockers on patients’ overall quality of life have yielded conflicting results. In the REDUCE-AMI study,^18^ long-term β-blocker use in patients after AMI with preserved left ventricular ejection fraction was not significantly different in individuals randomized to routine long-term β-blocker therapy as compared with individuals with no β-blocker use.

Proprotein convertase subtilisin/kexin type 9 inhibitors (PCSK9i) have recently emerged as promising therapeutic agents for lowering low-density lipoprotein cholesterol and reducing the risk of CV events. Moreover, preliminary evidence from randomized controlled trials suggests that PCSK9i may also offer beneficial effects for patients following VTE, with the most significant reductions in risk appearing over time, particularly beyond the first year of treatment.^12^ However, there is a lack of randomized controlled data supporting their efficacy and safety in conjunction with standard anticoagulation therapy. In a review paper from Dr Zuin *et al*. from Italy, the authors critically evaluate the existing evidence for the use of PCSK9i as a complementary therapy for VTE risk reduction. This review is commented on in an editorial from Dr Goto.

Over 230 million people are estimated to suffer from peripheral artery disease (PAD) globally.^22^ The burden of PAD is expected to grow dramatically as one of the most frequent first manifestations of CV disease in people with type 2 diabetes (T2D). There are few therapies that improve function and reduce symptoms in this population. Glucagon-like peptide-1 receptor agonists (GLP-1 RAs) have been shown to improve glycaemic control, reduce body weight, and reduce the risk of major adverse CV events, in people with atherosclerotic CV disease and T2D. STRIDE (NCT04560998) is a randomized, placebo-controlled, double-blind phase 3b trial evaluating 1 mg once-weekly subcutaneous semaglutide (GLP-1 RA) vs. placebo, in people with symptomatic PAD (Fontaine IIa claudication) and T2D. STRIDE has enrolled 792 participants with symptomatic PAD and T2D, frequent risk factors and comorbidities, and functional impairment. The trial will provide evidence for the functional outcomes with semaglutide in people with PAD and T2D.
